# Different measures of smoking exposure and mammographic density in postmenopausal Norwegian women: a cross-sectional study

**DOI:** 10.1186/bcr1782

**Published:** 2007-10-26

**Authors:** Yngve Bremnes, Giske Ursin, Nils Bjurstam, Inger T Gram

**Affiliations:** 1 Institute of Community Medicine, University of Tromsø, Tromsø, Norway; 2 Department of Preventive Medicine/Norris Comprehensive Cancer Center, University of Southern California Keck School of Medicine, Los Angeles, California, USA; 3 Department of Nutrition, University of Oslo, Norway; 4 Department of Radiology, Center for Breast Imaging, University Hospital of North Norway, Tromsø, Norway

## Abstract

**Background:**

Recent cohort studies have suggested an increased risk of breast cancer with long duration of smoking, and with smoking initiation before first birth. Cigarette smoking may have both carcinogenic effects and antiestrogenic effects on the breast tissue. We decided to examine the relationship between different measures of smoking exposure and mammographic density.

**Methods:**

Lifetime smoking history was collected through interview and questionnaires among 907 postmenopausal participants in the Tromsø Mammography and Breast Cancer study. The mammograms were obtained from the governmental Norwegian Breast Cancer Screening Program. Mammograms were classified according to the percentage and absolute mammographic densities using a previously validated computer-assisted method.

**Results:**

Sixty-five percent of the women reported having ever smoked cigarettes, while 34% were current smokers. After adjustment for age, age at first birth, parity, age at menopause, postmenopausal hormone therapy use, and body mass index, smoking was inversely associated with both measures of mammographic density (both trends *P *< 0.01). Both current smokers and former smokers had significantly lower adjusted mean percentage mammographic density compared with never smokers (*P *= 0.003 and *P *= 0.006, respectively). An inverse dose–response relationship with mammographic density was found between both the number of cigarettes and the number of pack-years smoked among current smokers. Current smokers who smoked 11 cigarettes or more daily had a 3.7% absolute (36% relative difference) lower percentage mammographic density compared with current smokers who smoked seven cigarettes or less daily (*P *= 0.008). When former smokers were stratified according to time since smoking cessation, we found that women who had stopped smoking less than 24 years ago had a significantly lower mean percentage mammographic density compared with never smokers (*P *< 0.001).

**Conclusion:**

We found modest inverse dose–response associations between numbers of cigarettes and of pack-years smoked and both measures of mammographic density among current smokers. Former smokers who had stopped smoking less than 24 years ago also had a statistically significantly lower mean percentage mammographic density when compared with never smokers. These findings are consistent with an antiestrogenic effect of cigarette smoking on the breast tissue.

## Introduction

Constituents in tobacco smoke may have carcinogenic effects on the breast tissue [1-3]. Tobacco smoking, however, may also have antiestrogenic effects that can reduce breast cancer risk [4,5]. These opposing effects may explain the overall inconsistent results from epidemiologic studies on the association between smoking and breast cancer risk [2,6-9]. Although most case–control studies do not find any positive associations [2,10], however, several recent cohort studies have indicated an increased breast cancer risk among women who are long-term smokers [11-14], and also among those who start to smoke before their first birth [12-17].

Mammographic density is one of the strongest independent risk factors for breast cancer [18,19], and possibly an intermediate marker for breast cancer [20]. Women with high mammographic density have a fourfold to sixfold increase in breast cancer risk compared with those with low mammographic density [18,19].

The results so far published on the association between smoking and mammographic density have also been conflicting [21-27]. One explanation for this may be that most studies have used crude (for example, ever/never) measures of smoking exposure.

The objective of the present cross-sectional study was to examine the relationship between cigarette smoking and mammographic density among postmenopausal women with a high smoking prevalence, according to different measures of smoking exposure.

## Materials and methods

### Study population

The Tromsø Mammography and Breast Cancer Study was conducted among postmenopausal women, aged 55–71 years, residing in the municipality of Tromsø, Norway, and attending the population-based Norwegian Breast Cancer Screening Program at the University Hospital of North Norway [28]. Women were recruited in spring 2001 and in spring 2002. After the women had undergone their screening mammograms, they were interviewed by a trained research nurse about reproductive and menstrual factors, previous history of cancer, current smoking status, and use of postmenopausal hormone therapy (HT) or other medications. The participants had their height measured to the nearest centimeter and their weight measured to the nearest half kilogram. Women had blood samples drawn, and each participant was subsequently given a questionnaire to be completed at home, eliciting information on demographics, additional menstrual and reproductive factors, lifetime smoking history, as well as lifestyle and dietary factors. All women signed an informed consent. The National Data Inspection Board and the Regional Committee for Medical Research Ethics approved the study. Altogether, 1,041 women were included in this cross-sectional study. This accounted for 70% of the women attending the Norwegian Breast Cancer Screening Program during the recruitment period.

We excluded 22 women because of a previously diagnosed (*n *= 16) or a newly diagnosed (*n *= 6) breast cancer, and one woman because of an ongoing chemotherapy treatment. Among the remaining 1,018 women, we were unable to retrieve mammograms on 11 women. We therefore obtained mammographic density readings on 1,007 women. More details are described elsewhere [28]. We further excluded three women because they were equivocal for menopausal status and excluded 97 women because of a missing smoking history, leaving 907 women for the analyses.

### Mammographic classifications

The women's left cranio-caudal mammogram was digitized using a Cobrascan CX-812 scanner (Radiographic Digital Imaging, Torrance, CA, USA) at a resolution of 150 pixels per inch. Percentage and absolute mammographic densities were determined by an experienced reader (GU) using the University of Southern California Madena computer-based threshold method, which has been described in detail and validated elsewhere [29]. Briefly, the method works as follows. The digitized mammographic image is viewed on a computer screen. A reader defines the total breast area using a special outlining tool. Next, the region of interest – excluding the pectoralis muscle, prominent veins, and fibrous strands – is defined. The computer software program assigns a pixel value of 0 to the darkest (black) shade in the image and a value of 255 to the lightest (white) shade, with shades of grey assigned to intermediate values. The reader then uses a tinting tool to apply a yellow tint to dense pixels with grey levels at or above some threshold *X *and a pixel value ≤ 255. The reader searches for the best threshold where all pixels ≥ *X *within the region of interest are considered to represent mammographic densities. The software estimates the total number of pixels and the number of tinted pixels within the region of interest. The absolute density represents the count of the tinted pixels within the region of interest. The percentage density, or the fraction (%) of the breast with densities, is the ratio of the absolute density to the total breast area multiplied by 100. The absolute density (measured in centimeters squared) was calculated as the number of tinted pixels within the region of interest divided by the number of pixels per centimeter squared.

The reader of the mammograms was blinded to characteristics of the study participants. We have previously shown a high intra-rater agreement for the reader in our study (Pearson correlation coefficient = 0.86) [28].

### Smoking assessments

The women were interviewed about their current smoking status. The self-administered questionnaire elicited additional information on the participant's lifetime smoking history. Women reporting never having smoked or never having been exposed to passive smoking were categorized as 'never active smokers'. We further categorized women who had never actively smoked but had been exposed to passive smoking at home or at the workplace as 'passive smokers'. Never active smokers and passive smokers were also grouped together as 'never smokers'. This group serves as the reference group in all analyses, if not specified otherwise. Current smokers and former smokers were grouped together as 'ever-smokers'. Pack-years were calculated as the number of cigarettes smoked daily divided by 20 and multiplied by the number of years smoked.

We categorized former smokers according to the time since smoking cessation (tertiles). We categorized current smokers according to their age at smoking initiation (tertiles), to the average number of cigarettes smoked per day (tertiles), to the number of years smoked (≤ 25 years, 26–40 years, 41+ years), to the number of pack-years smoked (tertiles), and parous women according to smoking initiation before or after first birth.

### Statistical analyses

Mammographic density was not normally distributed. Both the percentage and absolute mammographic densities were log-transformed to obtain approximate normal distributions. We used analysis of variance for an unbalanced design to study the associations between cigarette smoking and mammographic densities (Proc GLM; SAS Institute Inc., Cary, NC, USA).

Each of the following factors was evaluated as a potential confounder of the association between smoking and mammographic density: age at screening (continuous), age at menarche (continuous), age at menopause (continuous), number of children (continuous), age at first birth (continuous), years of education (continuous), family history of breast cancer in first-degree relatives (yes, no), alcohol intake (g/day, continuous), HT use (never used, past use, current use), and body mass index (BMI) (weight in kilogram divided by height in meters squared; continuous).

The variables of age at screening, number of children, HT use, and BMI have previously been found associated with mammographic density in this study population [28,30], and were always adjusted for as confounders in the multivariate analyses. We further identified those of the above listed variables that were associated with cigarette smoking in univariate analyses, and that also were significantly associated with mammographic density in the multivariate analyses. This procedure left the following factors in the final model: age at screening, age at first birth, number of children, age at menopause, HT use and BMI. Women with missing values were excluded from the analyses (<10%).

Reported trend-test *P *values correspond to analyses where the categories of cigarette smoking were treated as ordered variables. We tested for possible effect modification by analyzing the association between smoking and mammographic density stratified by the confounders, and by adding multiplicative interaction terms to the analysis of variance procedure.

The crude and adjusted mean mammographic density results were back-transformed, and are presented with 95% confidence intervals. Results were considered statistically significant if the two-sided *P *value was < 0.05. We conducted all statistical analyses using SAS^® ^9.1 for Windows (SAS Institute Inc.).

## Results

Altogether, 65% of the women were ever smokers. Among the 310 (34%) women who reported current smoking, 82% smoked daily. Altogether, 318 women reported to have never been smokers, among whom 73% reported to have been exposed to passive smoking at home or at the workplace.

Table 1 presents the distribution of selected characteristics according to smoking status. Current smokers were younger at screening, were younger at time of first birth, had less formal education, reached menopause at an earlier age, were leaner, and were more likely to have ever used oral contraceptives, when compared with never smokers (all *P *≤ 0.02).

**Table 1:** Characteristics of the study population (*n *= 907) by smoking status

Characteristic	Smoking status			*P *value^a^
	
	Current smokers (*n *= 310)	Former smokers (*n *= 279)	Never smokers (*n *= 318)	
Age at screening (years)	60.7 (4.4)	61.6 (4.5)	61.9 (4.6)	<0.001
Age at menarche (years)	13.3 (1.4)	13.3 (1.4)	13.3 (1.4)	0.47
Age at first birth^b ^(years)	22.3 (3.6)	22.7 (3.3)	23.5 (3.9)	<0.001
Number of children^b^	2.9 (1.2)	2.8 (1.1)	2.9 (1.4)	0.82
Education (years)	9.4 (3.2)	9.7 (3.4)	10.2 (3.6)	0.02
Age at menopause (years)	47.5 (5.5)	48.8 (4.8)	49.1 (4.8)	<0.001
Body mass index (kg/m^2^)	26.1 (4.4)	28.4 (5.1)	27.6 (4.7)	<0.001
Alcohol consumption^c ^(g/day)	3.8 (3.8)	4.0 (3.9)	3.4 (4.0)	0.36
Ever oral contraceptive use (%)	54.8	49.5	37.4	<0.001
Parous (%)	93.6	93.6	90.3	0.13
Ever postmenopausal hormone therapy use (%)	43.2	45.2	41.8	0.72
Breast cancer in first-degree relative (%)	6.8	9.3	9.8	0.18

Data presented as the mean (standard deviation) or as the frequency. ^a^Current smokers versus never smokers. ^b^Among parous women only.^c^Among alcohol drinkers only.

Former smokers also differed from never smokers, their data values being between current smokers and never smokers, with regard to age at first birth and ever oral contraceptive use (both *P *≤ 0.007). Former smokers had a significantly higher BMI when compared with never smokers (*P *= 0.04). Passive smokers were more likely to have ever used HT (*P *< 0.05), but were otherwise similar to never smokers in the other measured characteristics (results not shown).

Table 2 presents the median, the crude mean and the adjusted mean mammographic densities across smoking status. Both current smokers and former smokers had lower crude mean mammographic density compared with never smokers, according to both the percentage and the absolute mammographic densities. When the associations were adjusted for age at screening, for age at first birth, for number of children, for age at menopause, for HT use and for BMI, both current smokers and former smokers had a significantly lower adjusted mean percentage mammographic density compared with never smokers (*P *= 0.003 and *P *= 0.006, respectively). The magnitude of the difference in the mean percentage mammographic density between current smokers and never smokers was 2.2% absolute (23% relative) difference. A similar association was found between smoking status and absolute mammographic density (Table 2). These associations did not change materially when we excluded passive smokers from never smokers (results not shown), or when we excluded occasional smokers from current smokers (results not shown).

**Table 2:** Median, crude mean and adjusted mean percentage and absolute mammographic density by smoking status

Smoking status	*n*	Percentage mammographic density (%)			Absolute mammographic density (cm^2^)		
		
		Median (range)	Crude mean (95% confidence interval)	Adjusted mean (95% confidence interval)	Median (range)	Crude mean (95% confidence interval)	Adjusted mean (95% confidence interval)
Current smokers	310	9.4 (0–59.4)	8.0 (7.0–9.1)	7.3 (6.5–8.3)	13.7 (0–110.1)	10.7 (9.4–12.2)	10.4 (9.0–12.0)
Former smokers	279	7.7 (0–51.1)	6.7 (5.8–7.7)	7.5 (6.6–8.5)	11.9 (0–155.2)	9.7 (8.4–11.4)	10.6 (9.2–12.2)
Never smokers	318	11.9 (0–69.2)	9.7 (8.6–11.0)	9.5 (8.4–10.7)	17.1 (0–152.3)	14.0 (12.3–15.9)	13.7 (11.9–15.7)
*P *value^a^			0.03	0.003		0.004	0.005
*P *value^b^			<0.001	0.006		<0.001	0.01

Analyses are adjusted for age at screening, age at first birth, number of children, age at menopause, postmenopausal hormone therapy use, and body mass index. Reported means are back-transformed from log-transformed estimated means. ^a^Current smokers versus never smokers. ^b^Former smokers versus never smokers.

Current smokers on average smoked 10 cigarettes per day. The majority of current smokers had initiated smoking by the age of 20, and had been smoking for 39 years or more. Among those reporting to be former smokers, 71% had stopped smoking 10 years or more ago. Table 3 presents the association between different measures of smoking exposure and the percentage mammographic density among current smokers, and according to time since smoking cessation among former smokers. We found an inverse dose–response relationship between the number of cigarettes smoked daily and the percentage mammographic density among current smokers (*P *= 0.008), and this remained when the analysis was further restricted to current smokers who had smoked for 25 years or more (*P *= 0.01). Current smokers who smoked 11 cigarettes or more daily had a 3.7% absolute (36% relative difference) lower mean percentage mammographic density compared with current smokers who smoked seven cigarettes or less daily (*P *= 0.008). Furthermore, an inverse dose–response relationship was found between the number of pack-years and the percentage mammographic density among current smokers (*P *= 0.09). When former smokers were stratified according to time since smoking cessation, we found that women who had stopped smoking less than 24 years ago had a significantly lower mean percentage mammographic density compared with never smokers (*P *< 0.001). These associations were similar when absolute mammographic density was used as the outcome variable (results not shown).

**Table 3:** Adjusted mean percentage mammographic density according to smoking exposure among current smokers, and according to time since smoking cessation among former smokers

Smoking exposure	*n*	Adjusted mean percent mammographic density (95% confidence interval)	
		
		Current smokers	Former smokers
Number of cigarettes smoked per day			
≤ 7 cigarettes	87	10.3 (8.2–12.8)	
8–10 cigarettes	79	9.0 (7.0–11.5)	
≥ 11 cigarettes	77	6.6 (5.2–84)	
*P *trend		0.008	
Number of years smoked			
≤ 25 years	56	8.9 (6.8–11.8)	
26–40 years	92	8.8 (6.9–11.3)	
≥ 41 years	95	8.1 (6.5–10.1)	
*P *trend		0.57	
Number of pack-years smoked			
≤ 11 pack-years	82	10.0 (7.9–12.6)	
12–20 pack-years	85	8.5 (6.7–10.8)	
≥ 21 pack-years	75	7.5 (5.9–9.6)	
*P *trend		0.09	
Age at smoking initiation			
≥ 21 years	94	8.9 (7.1–11.1)	
18–20 years	114	8.4 (6.8–10.3)	
13–17 years	78	7.3 (5.8–9.3)	
*P *trend		0.26	
Smoking initiation before first birth^a^			
No	79	9.2 (7.2–11.8)	
Yes	186	7.8 (6.7–9.1)	
*P *value		0.24	
Time since smoking cessation			
≥ 24 years	89		8.5 (6.7–10.7)
12–23 years	89		5.2 (4.0–6.7)
≤ 11 years	88		6.5 (5.1–8.2)
*P *trend			0.12

Analyses are adjusted for age at screening, age at first birth, number of children, age at menopause, postmenopausal hormone therapy use, and body mass index. Reported means are back-transformed from log-transformed estimated means. ^a^Among parous women only.

## Discussion

This population-based cross-sectional study found an inverse association between smoking and percentage mammographic density among postmenopausal women, after adjustment for potential confounders. We also observed an inverse dose–response relationship among current smokers between both the numbers of cigarettes and pack-years smoked and the percentage mammographic density. Women who had stopped smoking less than 24 years ago had a significantly lower mean percentage mammographic density compared with never smokers These associations were similar when absolute mammographic density was used as the outcome variable.

The strengths of our study are the large sample size and that it was a part of a population-based screening program with a high attendance rate [31]. The reader of the mammograms was experienced and blinded to the characteristics of the women. We have previously shown a high intra-rater agreement for the reader in our study [28]. Further, we have assessed both relative and absolute measures of mammographic density as outcome variables, since they may be influenced differently by residual confounding by adiposity [32], and we found similar associations between smoking and mammographic density when we performed the analyses using the absolute mammographic density as the outcome variable. Another strength is that we have a large proportion of current smokers (34%) among the women in our study compared with previous studies among postmenopausal women, which all had less than 14% current smokers [21-24].

One limitation of our study is the possible misclassification of smoking exposure. Any misclassification of smoking would presumably be nondifferential with respect to mammographic density, and would therefore be expected to bias the results toward the null association. Another limitation is that the median mammographic density in our study is low and that mammographic density has a narrow range. In another study with a different reader, however, women from our study had significantly lower percentage mammographic density compared with Caucasians from Hawaii and Arizona [33].

In a study from the United States, Modugno and colleagues also found that current smokers had a significantly lower percentage mammographic density compared with noncurrent smokers. Their study, comprising 239 women aged 70 years or older, only had information about current smoking habits and therefore could not analyze the association in more detail [21].

Three other studies found no association between smoking and mammographic density among postmenopausal women [22-24]. In a British study with 406 women from Norfolk, Sala and colleagues found no significant association between smoking and high-risk Wolfe's parenchymal patterns among the 313 postmenopausal women in their study, even though current smokers had a significantly reduced odds ratio of having high-risk Wolfe's parenchymal patterns compared with never smokers when the analyses also included premenopausal women [22]. In an American study including 191 Hispanic women, Gapstur and colleagues found no association between smoking and mammographic density [23]. In another American study, Vachon and colleagues did not find any association between smoking duration and intensity and the percent mammographic density among 1,554 postmenopausal women, but an inverse association was found among the 346 premenopausal women in their study [24].

Mammographic density is an independent risk factor for breast cancer, and lower mammographic density could suggest lower risk for breast cancer [18,19]. One of the suggested mechanisms for how smoking might increases the risk of breast cancer is carcinogenic effects of constituents in tobacco smoke on the breast tissue [1-3]. It is possible that this suggested mechanism does not affect mammographic density, and thus would not influence mammographic density as measured in our study.

Conversely, the other suggested effect of smoking is believed to be an antiestrogenic effect [4,5,34]. Smoking has been shown to enhance the metabolism of estradiol to metabolites believed to have minimal peripheral estrogen activity, to increase estrogen binding by sex-hormone binding globulin and, because smokers tend to be leaner than nonsmokers, to lower the amount of estrogens derived from adipose tissue [4,5]. It has been shown that mammographic density may be intentionally manipulated by hormonal treatments such as tamoxifen or a gonadotropin-releasing hormone agonist [35,36]. A change in serum estrogen levels has also been shown to influence mammographic density [37]. The findings in the present study are consistent with an antiestrogenic effect of cigarette smoking on breast tissue, reflected in lower mammographic density among smokers.

We do not, however, believe our finding that current and former smokers have lower mean mammographic density when compared with never smokers will be reflected in a lower risk of breast cancer among smokers. The magnitudes of the difference in the mean percentage mammographic density between current smokers and never smokers were modest and may be due to residual confounding. We have previously shown that, in this study population, women using a continuous estrogen and progestin combination HT for 5 years or longer had a significantly higher percentage mammographic density compared with never HT users. The magnitude of that difference was substantially higher (7% absolute difference, 100% relative difference) than what we observed in the current study [30], suggesting that the effect of cigarette smoking may not be clinically relevant. Our finding that former smokers who had stopped smoking less than 24 years ago had statistically significantly lower mammographic density when compared with never smokers may partly be due to misclassification of current smokers as former smokers, as well as due to residual confounding. Boyd and colleagues, however, have proposed that the cumulative exposure to dense mammographic breast tissue could be represented by the area under the curve of the Pike and colleagues model for breast tissue ageing [38]. If there is an antiestrogenic effect of smoking, therefore, former smoking may have increased the age-related decrease in mammographic density.

## Conclusion

We found inverse associations between cigarette smoking and both the percentage and absolute mammographic densities among postmenopausal women in our cross-sectional study. We also observed a modest inverse dose–response association among current smokers between the amount smoked and the mammographic density. Also, former smokers who had stopped smoking less than 24 years ago had a statistically significantly lower mean percentage mammographic density when compared with never smokers. Our results are consistent with an antiestrogenic effect of smoking on the breast tissue, as measured by mammographic density.

## Abbreviations

BMI = body mass index; HT = postmenopausal hormone therapy.

## Competing interests

The authors declare that they have no competing interests.

## Authors' contributions

YB had full access to all data in the study, and takes responsibility for the integrity of the data and the accuracy of the data analyses. ITG conceived of the study, and had full access to all data. The classification of mammograms was performed by GU. YB, GU, NB, and ITG performed the analysis and interpretation of data. YB and ITG drafted the manuscript. Critical revision of the manuscript for important intellectual content was performed by YB, ITG, GU, and NB. All authors read and approved the final manuscript.
